# Reviewer Acknowledgement 2012

**DOI:** 10.1186/1471-2466-13-7

**Published:** 2013-03-02

**Authors:** Tim Shipley

**Affiliations:** 1 BioMed Central, 236 Gray's Inn Road, London, WC1X 8HB, United Kingdom

## Abstract

**Contributing reviewers:**

The editors of BMC Pulmonary Medicine would like to thank all our reviewers who have contributed to the journal in Volume 12 (2012).

## 

Raja Abboud

Canada

Richard Albert

United States of America

Namasivayam Ambalavanan

United States of America

Babak Amra

Iran

Wayne Anderson

United States of America

Claire Andrejak

France

Nick Antic

Australia

Rabbat Antoine

France

Raffaele Antonelli-Incalzi

Italy

Michele Anzidei

Italy

Inmaculada Arostegui

Spain

Simon Bacon

Canada

Parvaneh Baghaei Shiva

Iran

Marcel Baltzan

Canada

Albino Barraza-Villarreal

Mexico

Eric Bateman

South Africa

Jennifer Beane

United States of America

Jutta Beier

Germany

Scott Cameron Bell

Australia

Ronen Ben-Ami

Israel

Anne Bergeron

France

Nicole Beydon

France

Sangeeta Bhorade

United States of America

Mark Andrew Birrell

United Kingdom

Andrew Blann

United Kingdom

Alessandro Bodini

Italy

Francesco Bonella

Germany

Afshin Borhani Haghighi

Iran

Pierre-Olivier Bridevaux

Switzerland

Fraser Brims

United Kingdom

E Sherwood Brown

United States of America

Nigel Bruce

United Kingdom

Pierre-Regis Burgel

France

Charles Burger

United States of America

Michael Cabana

United States of America

Alberto Capelastegui

Spain

Chris Carlsten

Canada

Pierluigi Carratu

Italy

Alysson Carvalho

Brazil

Nola Cecins

Australia

Robert Centor

United States of America

Sally Chappell

United Kingdom

Niels Chavannes

Netherlands

Kazuo Chin

Japan

Young-Jae Cho

Korea, South

Seockhoon Chung

Korea, South

Catia Cilloniz

Spain

Michal Ciurzynski

Poland

Christian Clarenbach

Switzerland

Jennifer Cleland

United Kingdom

Stephen Michael Clift

United Kingdom

Marco Contoli

Italy

Richelle Cooper

United States of America

Antonio Corrado

Italy

Dirceu Costa

Brazil

Tim Counihan

Ireland

Peter Alan Coventry

United Kingdom

Jenette Creaney

Australia

Ernesto Crisafulli

Italy

Elliot Crouser

United States of America

Manuela De Sario

Italy

Lorenzo Del Sorbo

Canada

Christophe Delclaux

France

Marion Delcroix

Belgium

Pieter Depuydt

Belgium

Fabiano Di Marco

Italy

Gilda Diaz-Fuentes

United States of America

Duilio Divisi

Italy

Ilgaz Dogusoy

Turkey

Mark Dransfield

United States of America

Eric Duiverman

Netherlands

Marzia Duse

Italy

Georgie Eapen

United States of America

Tanja Effing

Australia

Nuha El Sharif

Israel

Souheil El-Chemaly

United States of America

Scott Evans

United States of America

Vicenç Falcó

Spain

Raymond Farah

Israel

Charles Feldman

South Africa

Anderson Ferreira

Brazil

Filippo Fimognari

Italy

Ellen Freiberger

Germany

Manabu Fujimoto

Japan

Leonello Fuso

Italy

Jinming Gao

China

Judith Garcia-Aymerich

Spain

Giancarlo Garuti

Italy

Ada Gastaldi

Brazil

Jose Gereda

Peru

Cristina Ghiciuc

Romania

Sudip Ghosh

United Kingdom

Irma Godoy

Brazil

Tomasz Golczewski

Poland

Michel Gonzalez

Switzerland

Renee Goodwin

United States of America

Alissa Greenberg

United States of America

Bogdan Grigoriu

Romania

Francisco Gude

Spain

Amund Gulsvik

Norway

Lida Hariri

United States of America

Naoki Hasegawa

Japan

Gilles Hejblum

France

Adam Hill

United Kingdom

Kylie Hill

Australia

Lan Ho-Pham

Viet Nam

Gary M. Hunninghake

United States of America

Masakazu Ichinose

Japan

Giuseppe Insalaco

Italy

Moshe Israeli

Israel

Patricia Jackson

United States of America

Matthew Jankowich

United States of America

Susan Janson

United States of America

Thomas Janssens

Belgium

Sanja Jelic

United States of America

Dennis Jensen

Canada

Zhi-Cheng Jing

China

Eun-Kyeong Jo

Korea, South

Paul Jones

United Kingdom

Manuela Joore

Netherlands

Tamilarasu Kadhiravan

India

John Kalbfleisch

United States of America

Edward Kaplan

United States of America

Alan Kaplan

Canada

Pinar Karaca-Mandic

United States of America

Dan Stieper Karbing

Denmark

Mahzuz Karim

United Kingdom

Wilfried Karmaus

United States of America

Steven Kawut

United States of America

Clive Kelly

United Kingdom

Klaus Kenn

Germany

Nicholas Kenyon

United States of America

Huib Adrianus Marie Kerstjens

Netherlands

Edward Kerwin

United States of America

Steven Kesten

United States of America

Farrah Kheramand

United States of America

Gopi Khilnani

India

Kozui Kida

Japan

Dalokay Kilic

Turkey

Victor Kim

United States of America

Greg King

Australia

Takeharu Koga

Japan

Konstantinos Kostikas

Greece

Antonia Koutsoukou

Greece

Rafal Krenke

Poland

Eva Kriegova

Czech Republic

Stefania La Grutta

Italy

Annabelle Lao

Philippines

Zorica Lazic

Serbia

Annemarie Lee

Australia

Adamantia Liapikou

Greece

Shu-Min Lin

Taiwan

Thomas Lincoln

United States of America

William Linn

United States of America

Thiago Lisboa

Spain

Carl Llor

Spain

Jose Luis Lopez-Campos

Spain

Fabrizio Luppi

Italy

Alex Mackay

United Kingdom

Toby Maher

United Kingdom

Nicola Makhoul

Israel

Mario Malerba

Italy

Patrick Mallia

United Kingdom

David Mannino

United States of America

Alessandro Marcon

Italy

Beatriz Martinez

Colombia

Joseph Massaro

United States of America

Stefan Matecki

France

Henryk Mazurek

Poland

David McAllister

United Kingdom

Eamon McGreal

United Kingdom

Maureen Meade

Canada

Keith C. Meyer

United States of America

Paul Mills

United States of America

Massimo Miniati

Italy

Oswald Monique

France

Frank Moosig

Germany

Aart Nicolaas Mudde

Netherlands

Desmond Murphy

Ireland

Patrick Murphy

United Kingdom

Lynne Murray

United Kingdom

Kristiaan Nackaerts

Belgium

Takahide Nagase

Japan

Luis Nannini

Argentina

Stefano Nava

Italy

Gabriele Nicolini

Italy

Ulrica G Nilsson

Sweden

Anna Nolan

United States of America

Imre Noth

United States of America

George Ntoumenopoulos

United Kingdom

Yasushi Obase

Japan

Toru Oga

Japan

Peter Pare

Canada

Sairam Parthasarathy

United States of America

Mical Paul

Israel

Dmitri Pechkovsky

Canada

Rodrigo Pedrosa

Brazil

Jesus Perez-Gil

Spain

Matthew Peters

Australia

Enrique Piacentini

Spain

Sara Piciucchi

Italy

Escribano Pilar

Spain

Leonardo Pinto

Brazil

Amanda Piper

Australia

Francesco Pistelli

Italy

Massimo Pistolesi

Italy

Celeste Porsbjerg

Denmark

Milo Puhan

United States of America

Bo Qiang

United States of America

Xiaoqun Qin

China

Philip Quanjer

Netherlands

Jennifer Quint

United Kingdom

Madeleine Rådinger

Sweden

Felix Ram

New Zealand

Salmo Raskin

Brazil

Emer Reeves

Ireland

Nichole Reisdorph

United States of America

Stephen Rennard

United States of America

Nicolas Roche

France

Eva Rönmark

Sweden

Russell Rosenberg

United States of America

Andras Rosztoczy

Hungary

Paola Rottoli

Italy

Bruce Rubin

United States of America

Lewis Rubin

United States of America

Gregg Ruppel

United States of America

Tarja Saaresranta

Finland

Eduardo Sada

Moldova

Mohsen Sadatsafavi

Canada

Sejal Saglani

United Kingdom

Qayyim Said

United States of America

Umadevi Sajjan

United States of America

Cesare Saltini

Italy

Adriana Salvaggio

Italy

Katerina Samara

Greece

Manuel Sanchez Luna

Spain

Scott Sands

United States of America

Mario Santini

Italy

Pierachille Santus

Italy

Bipin Savani

United States of America

Simone Scarlata

Italy

Andreas Schmiedl

Germany

Terence Seemungal

Trinidad and Tobago

Bulent Enis Sekerel

Turkey

David Serisier

Australia

Ajanta Sharma

India

Avi Shimony

Israel

Andrew Shorr

United States of America

Nikos Siafakas

Greece

Jodie Simpson

Australia

Richard Sinert

United States of America

Dave Singh

United Kingdom

Valérie Siroux

France

Peter Sly

Australia

Eric Snyder

United States of America

Lely Solari

Peru

Jinwoo Song

United States of America

Akshay Sood

United States of America

Banchob Sripa

Thailand

Jayne Standley

United States of America

Richard Stanford

United States of America

Frauke Stanke

Germany

Alistair Stewart

New Zealand

Kerstin Ström

Sweden

Cecilie Svanes

Norway

Dirk Taeger

Germany

Vin Tangpricha

United States of America

Claudio Tantucci

Italy

Ariel Tarasiuk

Israel

Robert Tarran

United States of America

Sadatomo Tasaka

Japan

Pierre Tattevin

France

Michiel Thomeer

Belgium

Bruce Thompson

Australia

Neil Thomson

United Kingdom

Rossella Tomaiuolo

Italy

Arzu Topeli

Turkey

Roberto Torchio

Italy

Josh Torgovnick

United States of America

Giorgio Treglia

Italy

Robert Tulloh

United Kingdom

Alice Turner

United Kingdom

Argyris Tzouvelekis

Greece

Bruce Uhal

United States of America

Françoise Van Bambeke

Belgium

Thys van der Molen

Netherlands

Kris Vanhaecht

Belgium

Jan Vercoulen

Netherlands

Andrew Vickers

United States of America

Fotios Vlastos

Greece

Claus Vogelmeier

Germany

Karin Vonbank

Austria

Claire Wainwright

Australia

Eugene Haydn Walters

Australia

Julia Walters

Australia

Jann-Yuan Wang

Taiwan

Tsu-Nai Wang

Taiwan

Mark Weatherall

New Zealand

Michael Weaver

United States of America

Christian Wejse

Denmark

Kristin Wickens

New Zealand

Renda Wiener

United States of America

Ed Wilson

United Kingdom

Sandra Wilson

United States of America

Tibor Wittmann

Hungary

Daniel Wolff

Germany

Ben-Quan Wu

China

Pinar Yildiz

Turkey

Ian Yang

Australia

Hongwei Yao

United States of America

Deborah Yates

Australia

Henry Yeager

United States of America

Panayiotis Yiallouros

Cyprus

Ozge Yilmaz

Turkey

In-Young Yoon

South Korea

Il Je Yu

South Korea

Carlos Zamarron

Spain

Glaucia Zanetti

Brazil

Ryan Zarychanski

Canada

Elias Zintzaras

Greece

## Pre-publication history

The pre-publication history for this paper can be accessed here:

http://www.biomedcentral.com/1471-2466/13/7/prepub

